# Filling Teeth

**Published:** 1870-06

**Authors:** 


### ARTICLE VIII.

### *Filling Teeth.*

#### CASE I.—PREPARATION OF THE CAVITY.

The right upper 2d molar has a shade of discoloration, showing on the posterior portion of its triturating surface.

The tooth lies closely to the 3d molar and has no appearance of decay near the grinding surface. The teeth are to be separated now with a wedge nearer the gum, and a delicate excavator does not discover a cavity near the grinding surface, but softened enamel is perceivod near the cervical portion, cutting like hard chalk.

The patient's mouth is small, if it was larger I would cut a dovetailed slot, with small hoes and hatches, from the *buccal* surface of the tooth, to the cavity. But in this case the mouth is to small for the proper manipulation of instruments, and therefore I proceed in the following manner:

With a crescent drill make a canal from the *grinding* surface of the tooth as directly upwards towards the cavity as possible. It must be *very* near the posterior approximal surface. Near the cervical portion the drill enters the softened dentine. I now cut out the thin portion of dentine between the canal which I have made and the posterior approximal surface of the tooth, leaving an opon canal, a section of which when finished must exhibit a "dovetail."

This open canal must correspond in diameter to that of the cavity of decay.

TO KEEP THE CAVITY DRY.

Wedge, to press back the gum at the cervical portion, or a bit of spunk crowded between the teeth at this point. A bit of spunk an inch square over the parotid duct of the same side held by a compressor. Sheet rubber laid over the lips and under the teeth as far back as convenient; over which is a thin white napkin to reflect light into the cavity; the gold contained in the plugging pliers will not get wet in being introduced, by any efforts of the patient in swallowing.

INSERTING THE GOLD.

Make two cylinders of soft, unadhesive gold, whose diameter shall each be a little more than half that of the cavity, and whose length shall be a little more than its antero posterior diameter. Place one of these cylinders within the cavity so as to allow one end to press against the adjoining tooth. With a foot-shaped plugger carry this cylinder about half way to the bottom of the cavity, then press it sidewise and carry the other cylinder to its side. With a little larger foot carry the whole of the gold *home*, and mallet it down. It can hardly be compacted too much. If well done the margin of the cavity nearest the alveolar process will be covered with gold and thoroughly protected. If the cylinders press well against the adjoining tooth they will remain *in situ* while more gold is added to them.

Add to the gold surface now made a thin layer of Morgan's plastic gold and condense thoroughly with very shallow and sharp instruments. Now cut No. 30 into narrow and short strips, and fill the remainder of the cavity, all the time keeping the plug against the adjoining tooth solidly. If this is done the teeth will be separated considerably by the time the operation is through, When near the surface use No. 60 in small square pieces and very small pointed pluggers. The gold may now be filed out which is pressed between the teeth and thoroughly polished.

POLISHING.

No plug can be well polished which is not dense. It must be uniformly dense near the surface. Deep serrated pluggers must not be used if a good surface is desired. It is impossible to fill the pits or obliterate them. They may be partly closed by condensing with a small smooth point for a long time.

Bring the surface down to the desired level with burrs and scrapers. If the gold has been properly condensed, it will cut like a solid piece of coin, and appear so also under the magnifying glass after burring it.

A deceptive polish may be made by nsing the burnisher, but it is only a thin film of gold which is thus made smooth, and which will in a few weeks wear off and show a rough surface.

After burring and scraping the plug, use pumice stone and a stick for quite a long while. Wash off the pumice from time to time and examine the margin. Form the latter to a continuous even line if possible, with a sharp excavator, or crescent drill, and then pumice it again, Pumice stone makes an *honest* and good finish; good enough in fact for all practical purposes for plugs that are not conspicuous. But we need not grudge a few minutes with chalk after the pumice. Prepared chalk on a soft stick for grinding surfaces, and chalk on chamois for approximal ones, will give a beautiful surface in a very short time, provided it has been sufficiently pumiced. A few drops of alcohol added makes it more agreeable in the mouth.

As it is not pleasant to the patient generally, to have these powders falling on the tongue, it is very proper to place a small napkin in such a way as to catch them, while polishing. CHASE.—*Missouri Dental Journal.*

---

